# Parallel comparison of risk factors between progression of organic stenosis in the coronary arteries and onset of acute coronary syndrome by covariance structure analysis

**DOI:** 10.1371/journal.pone.0173898

**Published:** 2017-03-16

**Authors:** Kazuo Ogawa, Kosuke Minai, Makoto Kawai, Toshikazu Tanaka, Tomohisa Nagoshi, Takayuki Ogawa, Michihiro Yoshimura

**Affiliations:** Division of Cardiology, Department of Internal Medicine, the Jikei University School of Medicine, Tokyo, Japan; Universitatsklinikum Freiburg, GERMANY

## Abstract

**Background:**

It is widely accepted that progression of organic stenosis in the coronary arteries and onset of acute coronary syndrome (ACS) are similar in the development of atherosclerosis. However, the extent of the association of each risk factor with the respective pathological conditions has not been fully elucidated.

**Objectives:**

We investigated the differences in risk factors between these conditions using a statistical procedure.

**Methods:**

The study population consisted of 1,029 patients with ischemic heart disease (IHD). We divided the study population into two groups (ACS and non-ACS) and by diseased vessels (organic stenosis). Covariance structure analysis was simultaneously performed in one equation model for determination and comparison of the risk factors for organic stenosis and ACS.

**Results:**

The analysis revealed that age (standardized regression coefficient, β: 0.206, *P* < 0.001), male gender (β: 0.126, *P* < 0.001), HbA1c level (β: 0.109, *P* < 0.001), HDL level (β: -0.109, *P* < 0.001) and LDL level (β: 0.127, *P* = 0.002) were significant for the advancement of organic stenosis. HDL level (β: 0100, *P* = 0.002) and MDA-LDL level (β: 0.335, *P* < 0.001) were significant for the onset of ACS, but age, HbA1c and LDL (*P* = NS or β < 0.1, respectively) were not. Among the risk factors, age, HbA1c and LDL were significantly more strongly associated with organic stenosis than ACS, while MDA-LDL was significantly more strongly associated with ACS than organic stenosis.

**Conclusions:**

The current statistical analysis revealed clear differences among the risk factors between the progression of organic stenosis and the onset of ACS. Among them, the MDA-LDL level should be considered to indicate a substantial risk of ACS.

## Introduction

There are several important risk factors for developing ischemic heart disease (IHD), including diabetes, hypertension, dyslipidemia, smoking and obesity [1–7]. Among the risk factors, cholesterol is a major component of the deposits that can narrow the coronary arteries. A high level of low-density lipoprotein (LDL) cholesterol in the blood may be due to an inherited condition or a diet high in saturated fats and cholesterol. Among cholesterol profiles, oxidized low-density lipoprotein (Ox-LDL) plays a pivotal role in the pathogenesis of atherosclerosis. Malondialdehyde-modified low-density lipoprotein (MDA-LDL) has been modified by MDA, leading to the production of a large amount of aldehyde when LDL degenerates and is oxidized [8]. It is known that the MDA-LDL level is elevated in patients with dyslipidemia and diabetes mellitus. Recently, MDA-LDL has been widely accepted as an important risk factor of IHD in general [9, 10].

It is important to take acute coronary syndrome (ACS) symptoms seriously as it is a life-threatening condition. Although ACS generally occurs in the background of atherosclerosis [11–13], ACS with no relevant narrowing of the coronary artery is a frequently occurring syndrome with a heterogeneous pathogenesis. Recently, the importance of inflammation and collagen metabolism has been recognized [14]. In addition, it is hypothesized that coronary spasm contributes to the onset of ACS to a greater or lesser degree in most cases [7, 15–17]. Nevertheless, patients occasionally do not experience ACS attacks despite the extensive progression of organic stenosis or calcification in the coronary arteries. Therefore, more precise mechanisms of atherosclerotic plaque initiation and progression, the methods by which plaques suddenly precipitate life-threatening thrombi, and the concepts of plaque burden, activity, and vulnerability should continue to be studied.

It is accepted that the progression of organic stenosis in the coronary arteries and the onset of ACS occur similarly in the development of atherosclerosis and that common risk factors therefore exist for both clinical conditions. However, the extent of each association to the progression of organic stenosis or ACS has not been elucidated. The risk factors might have different tendencies toward susceptibility to the progression of organic stenosis or onset of ACS.

A difficult aspect of this kind of study is how to design the study for simultaneous comparison of plural risk factors for respective pathological conditions. To the best of our knowledge, there is no report that uses such a complex study design. If possible, simultaneous analysis would be very helpful in gaining a deeper understanding of the characteristics of these risk factors. This new understanding will provide an efficient approach to the prevention and therapy of IHD in each individual.

Covariance structure analysis plays an important role in understanding how the relationship among observed variables might be generated by hypothesized latent variables in many areas. Covariance structure analysis is also useful for exploratory and explanatory factor analysis. When planning such an analysis, the input factors should be carefully selected. The path model, which is based on covariance structure analysis, should be proposed based on a well-defined concept and a clear direction of the study. Recently, we reported our research using covariance structure analysis in a tangible way [18].

We investigated the similarities and differences in the risk factors between the progression of organic stenosis in coronary arteries and the onset of ACS using covariance structure analysis in patients with IHD who were admitted consecutively to our institution. In this study, we aimed to clarify to which condition, organic stenosis or ACS, each risk factor is more likely linked and in particular to delineate more efficient prevention and therapy approaches for ACS in the future.

## Methods

### Study patients

The study population consisted of 1,029 patients with IHD who were admitted consecutively to our institution between 2012 and 2015. These patients underwent emergency cardiac catheterization upon admission or during the chronic phase. IHD was diagnosed based on the clinical features and examination findings, including the criteria of ischemic ECG changes and a blood test, following the universal definition of 2012[19]. In a practical sense, we diagnosed IHD in our institution as follows: in brief, (1) a history of chest pain, oppression, or discomfort lasting 20 min or longer. (2) A typical electrocardiographic change (i.e., ST segment elevation greater than 0.1 mV in at least 1 standard lead or 2 precordial leads, ST segment depression greater than 0.1 mV in at least 2 leads, or abnormal Q waves or T-wave inversions in at least 2 leads). (3) An increased serum level of the MB fraction of creatine kinase equivalent to more than twice the upper limit of the normal range or cardiac troponin T detected in blood using a TROPT^®^ sensitive rapid test strip. Patients without elevated biomarker values can be diagnosed as having unstable angina. According to these criteria, we could diagnose the study patients of acute coronary syndrome more precisely as having unstable angina pectoris (UAP), ST segment elevation myocardial infarction (STEMI), and non-ST segment elevation myocardial infarction (NSTEMI). In addition, stable angina pectoris was diagnosed when patients did not have angina at rest but had findings indicative of IHD based on exertional angina, ECG changes, and the morphology of the coronary arteries.

For the purpose of this study, we categorized the study patients in two ways. The first was to categorize the patients into ACS and non-ACS groups. The anamnestic history of a previous experience of ACS was irrelevant. The degree of coronary organic stenosis was also irrelevant in this method of classification.

The second categorization was based on the degree of organic stenosis in the coronary arteries. We counted the branch of the diseased vessels with organic stenosis of 75% and more; then, we divided the patients into four groups based on the number of diseased vessels: 0, 1, 2, or 3 vessels. The current and previous admitting status of ACS or non-ACS was irrelevant in this classification. Patients with coronary spasm induced by a provocation test with intracoronary injection of acetylcholine were categorized in the 0-vessel group if there was no organic stenosis after nitroglycerin administration.

Patients who previously received coronary intervention and/or coronary artery bypass grafting were excluded from the study population. This study was approved by the ethics committee of the Jikei University School of Medicine (study protocol: 24–150[6916]), and we complied with the routine ethical regulations of our institution as follows: this is a retrospective study and informed consent could not be obtained from each patient. Instead of informed consent from each patient, we publicly posted a notice regarding the study design and contact information at a publicly known place in our institution.

### Blood sampling and measurement of biochemical examination

Blood sampling was conducted for every patient with IHD during cardiac catheterization. Serum biochemical analyses were performed in a central laboratory in our hospital.

### Statistical analysis

Continuous variables were expressed as the means ± standard deviation (SD) or the median. Statistical analyses were performed using IBM SPSS Statistics version 23.0 (SPSS Inc., Chicago, IL, USA). Multiple regression analysis was adopted on an as-needed basis. Path analysis based on covariance structure analysis was used to investigate the relationship among clinical factors in this study population and particularly to identify probable causal effects in organic stenosis or ACS. Path analysis was performed using IBM SPSS AMOS version 23 (Amos Development Corporation, Meadville, PA, USA). The obtained structural equation models were tested and confirmed at the significance level for *P* values of <0.05. The implementation procedures of covariance structure analysis have been described previously [18].The causality model defines certain hierarchical regression models that compare clinical factors in organic stenosis and ACS. When obtaining critical ratios for differences between parameters, AMOS supplies a matrix with a row and column for each parameter of the model. Each off-diagonal entry in the provides gives a statistic for testing the hypothesis that two model parameters are equal in the population.

## Results

### Study patient characteristics

Among the 1,029 patients with IHD, 310 patients were diagnosed as ACS and 719 patients as non-ACS at admission. Of the total 1,029 patients, 252 patients had 0-vessel disease, 453 patients had 1-vessel disease, 212 patients had 2-vessel disease, and 112 patients had 3-vessel disease. Additional characteristics of the non-ACS and ACS groups are shown in Table 1.

**Table 1:** Patient characteristics of non-ACS and ACS groups.

N = 1,029	Non-ACS	ACS
Number of patients (%)	719 (69.9)	310 (30.1)
Age	65.5 ± 10.5	64.5 ± 13.1
Male, gender (%)	621 (86.4)	244 (78.7)
BMI, kg/m^2^	24.6 ± 3.7	24.3 ± 4.2
Smoker (%)	180 (25.0)	95 (30.6)
HbA1c, %	6.3 ± 1.0	6.3 ± 1.3
LDL, mg/dℓ	103.7 ± 30.2	117.9 ± 39.2*
HDL, mg/dℓ	51.8 ± 15.3	53.6 ± 17.2
MDA-LDL, U/L	120.3 ± 41.0	154.2 ± 69.5*
Underlying diseases		
Diabetes mellitus (%)	307 (42.7)	109 (35.2)*
Hypertension (%)	543 (75.5)	230 (74.1)
Dyslipidemia (%)	531 (73.9)	222 (71.6)
Diseased vessel (%)		
0VD	180 (25.0)	72 (23.2)
1VD	310 (43.1)	143 (46.1)
2VD	152 (21.1)	60 (19.3)
3VD	77 (10.7)	35 (11.3)
UAP (%)		168 (54.2)
NSTEMI (%)		42 (13.5)
STEMI (%)		100 (32.3)
Medication (%)		
Statin	385 (53.5)	79 (25.5)*
CCB	416 (57.9)	117 (37.7)*
β-blocker	266 (37.0)	34 (11.0)*
ACE/ARB	175 (24.3)	108 (34.3)*
Antiplatelet	226 (31.4)	85 (27.4)

*: *P* < 0.05: vs. non-ACS group.

ACS = acute coronary syndrome; BMI = body mass index; HbA1c = hemoglobin A1c; LDL = low-density lipoprotein cholesterol; HDL = high-density lipoprotein cholesterol; MDA-LDL = malondialdehyde-modified low-density lipoprotein; 0VD = 0-vessel disease; 1VD = single-vessel disease; 2VD = double-vessel disease; 3VD = triple-vessel disease; UAP = unstable angina pectoris; NSTEMI = non-ST segment elevation myocardial infarction; STEMI = ST segment elevation myocardial infarction; CCB = calcium channel blocker; β-blocker = beta blocker; ACE-I = angiotensin converting enzyme inhibitor; ARB = angiotensin receptor blocker

### Multivariate analysis for determination of risk factors of the onset of ACS or the progression of CAD

The several potential risk factors, which were listed as candidates based on previous information, were included in the analysis; age, gender, HbA1c, high-density lipoprotein (HDL), LDL, MDA-LDL, smoking, hypertension and body mass index (BMI) were used as dependent variables (Tables 2 and 3). We adopted multiple logistic regression analysis by using 0 as the independent value for non-ACS and 1 for ACS. As a result, multivariate analysis revealed that male gender, high HDL and high MDA-LDL represented the risks for ACS (Table 2). Multiple linear regression analysis was adapted for determination of the diseased vessels. As a result, multivariate analysis revealed that age, male gender, HbA1c, low HDL, high LDL and hypertension represented risks or CAD (Table 3). Thus, MDA-LDL would represent a risk for ACS but not for organic stenosis. In contrast, HbA1c represented a risk for organic stenosis but not for ACS. However, these respective multivariate analyses would still be inadequate for interpreting the risk factors correctly because the factors are expected to confound each other and because it is challenging to make the characteristics of each risk factor explicit using the respective equation models (i.e., not using a unified equation model).

**Table 2:** Multivariate analysis for determination of ACS.

	Regression coefficients	Standard error	*P* value	Odds ratio
Gender	-0.47	0.20	0.02	0.63
HDL	0.01	0.01	0.01	1.01
MDALDL	0.01	0.00	0.00	1.01

Objective variable: ACS or non-ACS

Explanatory variable: age, gender, HbA1c, HDL, LDL, MDALDL, smoking, hypertension, BMI, Non-significant variables: age, HbA1c, LDL, smoking, hypertension, BMI

ACS = acute coronary syndrome; HbA1c = hemoglobin A1c; HDL = high-density lipoprotein cholesterol; LDL = low-density lipoprotein cholesterol; MDA-LDL = malondialdehyde-modified low-density lipoprotein; BMI = body mass index

**Table 3:** Multivariate analysis for diseased vessels.

	Regression coefficients	Standard error	Test statistic	*P* value
Age	0.012	0.002	5.54	<0.001
Gender	0.235	0.061	3.825	<0.001
HbA1c	0.091	0.02	4.571	<0.001
HDL	-0.005	0.001	-3.186	0.001
LDL	0.006	0.001	6.339	<0.001
Hypertension	0.102	0.05	2.03	0.043

Objective variable: the number of diseased vessels

Explanatory variable: age, gender, HbA1c, HDL, LDL, MDALDL, smoking, hypertension, BMI

Non-significant variables: MDA-LDL, smoking, BMI

HbA1c = hemoglobin A1c; HDL = high-density lipoprotein cholesterol; LDL = low-density lipoprotein cholesterol; MDA-LDL = malondialdehyde-modified low-density lipoprotein; BMI = body mass index

### Concept of Path model A

**Fig 1:**
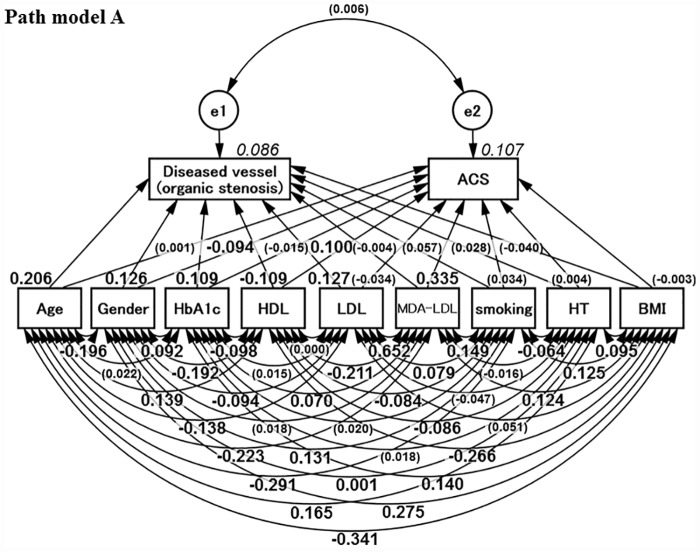
Proposed Path model A. The path has estimates of standardized regression weights and estimates of correlations among exogenous variables. The variable (given in parentheses) means “not statistically significant.” HbA1c = hemoglobin A1c; HDL-C = high-density lipoprotein cholesterol; LDL = low-density lipoprotein; MDA-LDL = malondialdehyde-modified LDL; HDL = high-density lipoprotein; HT = hypertension; BMI = body mass index.

The proposed path model is shown in Fig 1. Logically, the risk factors are expected to potentially confound each other; the association between two factors is linked by the two-way arrows. Paths between variables are drawn from independent to dependent variables, with a directional arrow for every regression model, namely, from age, gender, HbA1c, HDL,LDL, MDA-LDL, smoking, hypertension, and BMI to the diseased vessels and to ACS. In the analysis, the two-way arrow between the degree of organic stenosis and ACS (namely, e1–e2) was of intrinsic importance. In the path model, the number of diseased vessels was counted as 0, 1, 2 or 3, respectively.

### Results of Path model A: Estimation of regression weights in organic stenosis

Table 4 shows the estimates of regression weights and estimates of standardized regression weights. The analysis using the path model to examine the risk factors for the advancement of organic stenosis revealed that age (standardized regression coefficient; β: 0.206, *P* < 0.001), male gender (β: 0.126, *P* < 0.001), HbA1c (β: 0.109, *P* < 0.001) and LDL ((β: 0.127, *P* < 0.001) were the significant factors. Note that MDA-LDL is not statistically correlated with advancement of organic stenosis (*P* = NS).

**Table 4:** Path model A: Estimates of regression weight and standard regression weight.

Clinical Factor		Estimate	Standard error	Test statistic	P Value	Standard regression coefficient
ACS (R^2^ = 0.107)	<--Age	0.000	0.001	0.016	0.987	0.001
	<--Gender	-0.101	0.039	-2.563	0.010	-0.081
	<--HbA1c	-0.006	0.012	-0.491	0.624	-0.015
	<--HDL	0.003	0.001	3.128	0.002	0.100
	<--LDL	0.000	0.001	-0.847	0.397	-0.034
	<--MDALDL	0.003	0.000	8.166	<0.001	0.335
	<--Smoking	0.035	0.032	1.099	0.272	0.034
	<--HT	0.004	0.032	0.118	0.906	0.004
	<--BMI	-0.004	0.004	-0.906	0.365	-0.030
Diseased Vessel (R^2^ = 0.086)	<--Age	0.017	0.003	5.985	<0.001	0.206
	<--Gender	0.319	0.080	3.959	<0.001	0.126
	<--HbA1c	0.090	0.025	3.591	<0.001	0.109
	<--HDL	-0.006	0.002	-3.381	<0.001	-0.109
	<--LDL	0.003	0.001	3.160	0.002	0.127
	<--MDALDL	0.000	0.001	-0.104	0.917	-0.004
	<--Smoking	0.120	0.066	1.828	0.068	0.057
	<--HT	0.061	0.066	0.922	0.357	0.028
	<--BMI	-0.010	0.008	-1.186	0.236	-0.040

ACS = acute coronary syndrome; HbA1c = hemoglobin A1c; HDL = high-density lipoprotein cholesterol; LDL = low-density lipoprotein cholesterol; MDA-LDL = malondialdehyde-modified low-density lipoprotein; HT = hypertension; BMI = body mass index

### Results of Path model A: Estimation of regression weights in ACS

As also shown in Table 4, the analysis using the path model to examine the risk factors for ACS revealed that gender (β: −0.081, *P* = 0.010), HDL (β: 0.100, *P* = 0.002) and MDA-LDL (β: 0.355, *P* < 0.001) were the significant factors. Considering the power of β, it is of note that the significance of MDA-LDL was substantially higher than that of other factors. This finding was statistically confirmed by critical ratios for the differences between parameters; MDA-LDL was more strongly associated than any other parameter with ACS (*P* < 0.01, respectively), which was examined in the matrix for each parameter of this model. (The full matrix is not shown due to size constraints.)

### Results of Path model A: Association among relative risk factors

Table 5 shows the results of estimates of covariance among exogenous variables and estimates of correlations among exogenous variables. Among them, the association between LDL and MDA-LDL was prominent (β; 0.652, *P <* 0.001). The association between organic stenosis and ACS (namely, e1–e2) was of importance; the *P* value was not significant (*P* = 0.851).

**Table 5:** Path model A: Association between relative risk factors.

Clinical factor			Estimate	Standard error	Test statistic	*P* value	Standard regression coefficient
BMI	<-->	Age	-14.835	1.435	-10.335	<0.001	-0.341
	<-->	Gender	0.386	0.045	8.503	<0.001	0.275
	<-->	HbA1c	0.601	0.136	4.421	<0.001	0.140
	<-->	HDL	-16.225	1.971	-8.233	<0.001	-0.266
	<-->	LDL	6.604	4.047	1.632	0.103	0.051
	<-->	MDALDL	25.504	6.455	3.951	<0.001	0.124
	<-->	Smoking	0.211	0.053	3.963	<0.001	0.125
	<-->	HT	0.158	0.052	3.044	0.002	0.095
HT	<-->	Age	0.809	0.155	5.209	<0.001	0.165
	<-->	Gender	0.000	0.005	0.039	0.969	0.001
	<-->	HbA1c	0.009	0.015	0.580	0.562	0.018
	<-->	HDL	-0.589	0.216	-2.733	0.006	-0.086
	<-->	LDL	-0.690	0.456	-1.513	0.130	-0.047
	<-->	MDALDL	-0.371	0.722	-0.513	0.608	-0.016
	<-->	Smoking	-0.012	0.006	-2.045	0.041	-0.064
Smoking	<-->	Age	-1.461	0.163	-8.946	<0.001	-0.291
	<-->	Gender	0.021	0.005	4.164	<0.001	0.131
	<-->	HbA1c	0.010	0.016	0.632	0.527	0.020
	<-->	HDL	-0.591	0.221	-2.678	0.007	-0.084
	<-->	LDL	1.180	0.468	2.521	0.012	0.079
	<-->	MDALDL	3.543	0.748	4.739	<0.001	0.149
MDALDL	<-->	Age	-135.825	19.454	-6.982	<0.001	-0.223
	<-->	Gender	0.357	0.612	0.583	0.560	0.018
	<-->	HbA1c	4.198	1.887	2.225	0.026	0.070
	<-->	HDL	-179.680	27.197	-6.607	<0.001	-0.211
	<-->	LDL	1179.820	67.406	17.503	<0.001	0.652
LDL	<-->	Age	-53.013	12.093	-4.384	<0.001	-0.138
	<-->	Gender	-1.163	0.388	-3.001	0.003	-0.094
	<-->	HbA1c	0.583	1.188	0.491	0.624	0.015
	<-->	HDL	-0.088	16.792	-0.005	0.996	0.000
HDL	<-->	Age	25.174	5.700	4.417	<0.001	0.139
	<-->	Gender	-1.119	0.185	-6.042	<0.001	-0.192
	<-->	HbA1c	-1.760	0.562	-3.130	0.002	-0.098
HbA1c	<-->	Age	0.279	0.399	0.699	0.484	0.022
	<-->	Gender	0.038	0.013	2.940	0.003	0.092
Gender	<-->	Age	-0.814	0.132	-6.155	<0.001	-0.196
e1	<-->	e2	0.002	0.012	0.188	0.851	0.006

HbA1c = hemoglobin A1c; HDL = high-density lipoprotein cholesterol; LDL = low-density lipoprotein cholesterol; MDA-LDL = malondialdehyde-modified low-density lipoprotein; HT = hypertension; BMI = body mass index

### Results of Path model A: Critical ratios of differences between parameters (risk factors associated with organic stenosis or ACS)

Among the risk factors, the critical ratios for differences between parameters were examined via the matrix. Age, LDL, and HbA1c were more strongly associated with organic stenosis than ACS (*P* < 0.01, respectively); in contrast, the MDA-LDL level was more strongly associated with ACS than diseased vessels (*P* < 0.01). (The full matrix is not shown due to size constraints.)

### Concept of Path model B and the results

**Fig 2:**
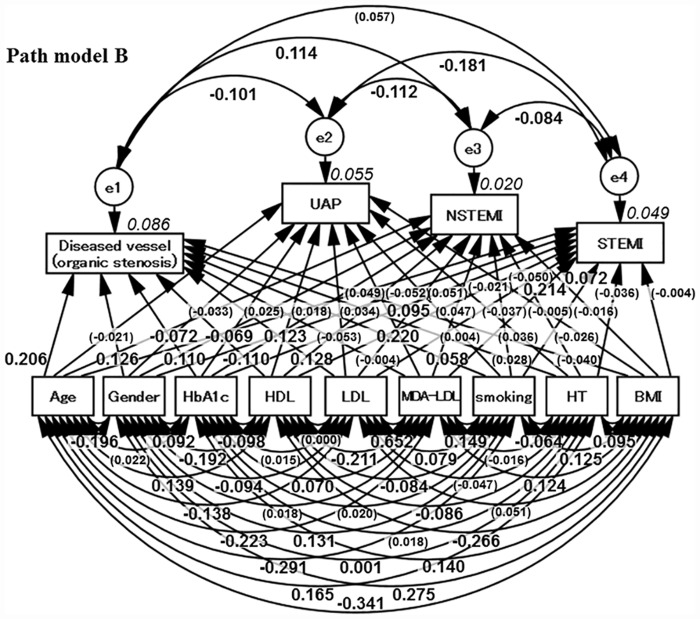
Proposed Path model B (sub-group analysis of acute coronary syndrome). The path has estimates of standardized regression weights and estimates of correlations among exogenous variables. The variable (given in parentheses) means “not statistically significant.” UAP = unstable angina pectoris; STEMI = ST segment elevation myocardial infarction; NSTEMI = non-ST segment elevation myocardial infarction; HbA1c = hemoglobin A1c; HDL-C = high-density lipoprotein cholesterol; LDL = low-density lipoprotein; MDA-LDL = malondialdehyde-modified LDL; HDL = high-density lipoprotein; HT = hypertension; BMI = body mass index.

Next, Path model B was developed in a similar way to Path model A by dividing the ACS patients into three groups: unstable angina pectoris (UAP), non-ST segment elevation myocardial infarction (NSTEMI) and ST segment elevation myocardial infarction (STEMI) groups, as shown in Fig 2. MDA-LDL was associated with UAP and STEMI. There was, however, no statistically significant association between MDA-LDL and NSTEMI (Table 6).

**Table 6:** Path model B: Estimates of regression weight and standard regression weight.

Clinical Factor		Estimate	Standard error	Test statistic	P Value	Standard regression coefficient
Diseased Vessel (R^2^ = 0.086)	<--Age	0.017	0.003	5.993	<0.001	0.206
	<--Gender	0.319	0.080	3.965	<0.001	0.126
	<--HbA1c	0.090	0.025	3.603	<0.001	0.110
	<--HDL	-0.006	0.002	-3.388	<0.001	-0.110
	<--LDL	0.003	0.001	3.166	0.002	0.128
	<--MDALDL	0.000	0.001	-0.106	0.916	-0.004
	<--Smoking	0.120	0.066	1.831	0.067	0.058
	<--HT	0.061	0.066	0.923	0.356	0.028
	<--BMI	-0.010	0.008	-1.188	0.235	-0.040
UAP (R^2^ = 0.055)	<--Age	-0.001	0.001	-0.603	0.547	-0.021
	<--Gender	-0.072	0.033	-2.209	0.027	-0.072
	<--HbA1c	-0.023	0.010	-2.231	0.026	-0.069
	<--HDL	0.003	0.001	3.739	<0.001	0.123
	<--LDL	-0.001	0.000	-1.294	0.196	-0.053
	<--MDALDL	0.002	0.000	5.196	<0.001	0.220
	<--Smoking	0.004	0.027	0.135	0.892	0.004
	<--HT	0.031	0.027	1.155	0.248	0.036
	<--BMI	-0.003	0.003	-0.756	0.449	-0.026
NSTEMI (R^2^ = 0.020)	<--Age	-0.001	0.001	-0.939	0.348	-0.033
	<--Gender	0.013	0.018	0.753	0.451	0.025
	<--HbA1c	0.003	0.006	0.581	0.561	0.018
	<--HDL	0.000	0.000	1.017	0.309	0.034
	<--LDL	0.001	0.000	2.277	0.023	0.095
	<--MDALDL	0.000	0.000	1.094	0.274	0.047
	<--Smoking	-0.017	0.015	-1.137	0.256	-0.037
	<--HT	-0.002	0.015	-0.163	0.870	-0.005
	<--BMI	-0.001	0.002	-0.443	0.657	-0.016
STEMI (R^2^ = 0.049)	<--Age	0.001	0.001	1.409	0.159	0.049
	<--Gender	-0.042	0.026	-1.611	0.107	-0.052
	<--HbA1c	0.014	0.008	1.650	0.099	0.051
	<--HDL	0.000	0.001	-0.640	0.522	-0.021
	<--LDL	0.000	0.000	-1.207	0.228	-0.050
	<--MDALDL	0.001	0.000	5.052	<0.001	0.214
	<--Smoking	0.048	0.021	2.251	0.024	0.072
	<--HT	-0.025	0.022	-1.147	0.251	-0.036
	<--BMI	0.000	0.003	-0.116	0.908	-0.004

ACS = acute coronary syndrome; UAP = unstable angina pectoris; NSTEMI = non-ST segment elevation myocardial infarction; STEMI = ST segment elevation myocardial infarction; HbA1c = hemoglobin A1c; HDL = high-density lipoprotein cholesterol; LDL = low-density lipoprotein cholesterol; MDA-LDL = malondialdehyde-modified low-density lipoprotein; HT = hypertension; BMI = body mass index

### Concept of Path model C and the results

**Fig 3:**
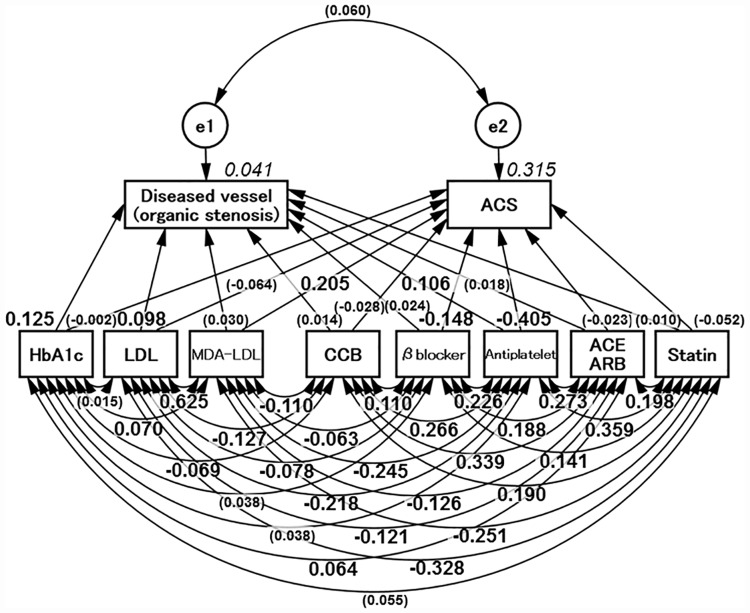
Proposed Path model C (Potential influence of drugs). The path has estimates of standardized regression weights and estimates of correlations among exogenous variables. The variable (given in parentheses) means “not statistically significant.” HbA1c = hemoglobin A1c; HDL-C = high-density lipoprotein cholesterol; LDL = low-density lipoprotein; MDA-LDL = malondialdehyde-modified LDL; HDL = high-density lipoprotein; HT = hypertension; BMI = body mass index. ACE = angiotensin-converting enzyme; ARB = angiotensin II receptor blocker; CCB = calcium channel blocker.

Finally, Path model C was proposed, as shown in Fig 3. The possible drug effects on the current study were taken into consideration. The analysis revealed that LDL, age and HbA1c were significant for the advancement of organic stenosis. The MDA-LDL level was significant for the onset of ACS. Thus, the main results in Path model C were not affected by the applied medication and were similar to those in Path model A. In this path model, only as a guide, antiplatelet therapy was effective in suppressing both ACS and organic stenosis (Tables 7 and 8).

**Table 7:** Path model C: Estimates of regression weight and standard regression weight.

Clinical Factor		Estimate	Standard error	Test statistic	P Value	Standard regression coefficient
ACS (R^2^ = 0.315)	<--HbA1c	-0.001	0.011	-0.084	0.933	-0.002
	<--LDL	-0.001	0.000	-1.819	0.069	-0.064
	<--MDALDL	0.002	0.000	5.941	<0.001	0.205
	<--CCB	-0.026	0.026	-1.005	0.315	-0.028
	<--β-blocker	-0.149	0.027	-5.528	<0.001	-0.148
	<--Antiplatelet	-0.388	0.028	-13.768	<0.001	-0.405
	<--ACE/ARB	-0.021	0.026	-0.821	0.411	-0.023
	<--Statin	-0.048	0.027	-1.777	0.076	-0.052
Diseased Vessel (R^2^ = 0.041)	<--HbA1c	0.103	0.026	4.026	<0.001	0.125
	<--LDL	0.003	0.001	2.365	0.018	0.098
	<--MDALDL	0.001	0.001	0.741	0.459	0.030
	<--CCB	0.027	0.062	0.430	0.667	0.014
	<--β-blocker	0.048	0.064	0.752	0.452	0.024
	<--Antiplatelet	0.205	0.067	3.042	<0.001	0.106
	<--ACE/ARB	0.032	0.062	0.521	0.603	0.018
	<--Statin	0.019	0.064	0.305	0.760	0.010

ACS = acute coronary syndrome; HbA1c = hemoglobin A1c; LDL = low-density lipoprotein cholesterol; MDA-LDL = malondialdehyde-modified low-density lipoprotein; CCB = calcium channel blocker; β-blocker = beta blocker; ACE = angiotensin converting enzyme inhibitor; ARB = angiotensin receptor blocker

**Table 8:** Path model C: Association between relative risk factors.

Clinical factor			Estimate	Standard error	Test statistic	P value	Standard regression coefficient
Statin	<-->	HbA1c	0.031	0.018	1.771	0.077	0.055
	<-->	LDL	-5.509	0.552	-9.981	<0.001	-0.328
	<-->	MDALDL	-6.691	0.857	-7.806	<0.001	-0.251
	<-->	CCB	0.047	0.008	5.99	<0.001	0.19
	<-->	β-blocker	0.032	0.007	4.465	<0.001	0.141
	<-->	Antiplatelet	0.086	0.008	10.838	<0.001	0.359
	<-->	ACE/ARB	0.049	0.008	6.238	<0.001	0.198
ACE/ARB	<-->	HbA1c	0.036	0.018	2.05	0.04	0.064
	<-->	LDL	-2.039	0.531	-3.841	<0.001	-0.121
	<-->	MDALDL	-3.365	0.842	-3.997	<0.001	-0.126
	<-->	CCB	0.085	0.008	10.303	<0.001	0.339
	<-->	β-blocker	0.043	0.007	5.91	<0.001	0.188
	<-->	Antiplatelet	0.065	0.008	8.437	<0.001	0.273
Antiplatelet	<-->	HbA1c	0.02	0.017	1.213	0.225	0.038
	<-->	LDL	-3.524	0.517	-6.823	<0.001	-0.218
	<-->	MDALDL	-6.276	0.823	-7.621	<0.001	-0.245
	<-->	CCB	0.064	0.008	8.255	<0.001	0.266
	<-->	β-blocker	0.049	0.007	7.082	<0.001	0.226
β-blocker	<-->	HbA1c	0.019	0.016	1.216	0.224	0.038
	<-->	LDL	-1.194	0.481	-2.485	0.013	-0.078
	<-->	MDALDL	-1.546	0.761	-2.031	0.042	-0.063
	<-->	CCB	0.025	0.007	3.493	<0.001	0.11
CCB	<-->	HbA1c	-0.039	0.018	-2.203	0.028	-0.069
	<-->	LDL	-2.14	0.531	-4.03	<0.001	-0.127
	<-->	MDALDL	-2.952	0.84	-3.515	<0.001	-0.11
MDALDL	<-->	HbA1c	4.21	1.887	2.231	0.026	0.07
	<-->	LDL	1179.82	67.406	17.503	<0.001	0.652
HbA1c	<-->	LDL	0.578	1.188	0.487	0.627	0.015
e1	<-->	e2	0.021	0.011	1.922	0.055	0.06

HbA1c = hemoglobin A1c; LDL = low-density lipoprotein cholesterol; MDA-LDL = malondialdehyde-modified low-density lipoprotein; CCB = calcium channel blocker; β-blocker = beta blocker; ACE = angiotensin converting enzyme inhibitor; ARB = angiotensin receptor blocker

## Discussion

In this study, we investigated the differences in the risk factors between the progression of organic stenosis and the onset of ACS using covariance structure analysis. First, this study showed that the link between the organic stenosis and ACS groups (e1–e2) was not statistically significant, as shown in Table 5. In addition, there were substantial differences in the contribution of risk factors to organic stenosis or ACS. HbA1c and LDL were strong risk factors for the advancement of organic stenosis, whereas MDA-LDL showed a substantial risk for ACS. The contribution of MDA-LDL to organic stenosis was not significant in this study. Previous findings showed MDA-LDL to be risk factor of IHD in general [8–10]. However, an unambiguous strong association between MDA-LDL and ACS had not been previously reported, and the current study is the first to clearly show, in a parallel comparison, the distinct effect of MDA-LDL on the onset of ACS rather than on the advancement of organic stenosis.

The precise mechanisms of the close correlation between MDA-LDL and ACS are unknown at present, although it is probable that endothelial dysfunction is the core mechanism. Endothelial dysfunction is widely accepted as the earliest event in atherosclerosis [20]. Ox-LDL is known to enhance the expression of pro-inflammatory molecules, leading to monocyte recruitment in the vessel wall. Ox-LDL is cytotoxic to endothelial cells via the generation of free radicals and impairs nitric oxide synthase gene expression and its activity. Ox-LDL triggers the activation of an inflammatory signaling pathway and increases gene expression and the activity of matrix metalloproteinases in endothelial cells [21]. It is also notable that Ox-LDL-associated endothelial dysfunction induces coronary spasms, even though LDL itself is not necessarily associated with coronary spasms [7].

The relatively strong risk factors associated with organic stenosis were age, HbA1c and LDL. The mechanism of the strong contribution of HbA1c to the advancement of organic stenosis, but not to ACS, is of interest. We speculate that diabetes contributes to the vascular smooth muscle cell (VSMC) phenotype. VSMCs can perform both contractile and synthetic functions, which are associated with and characterized by changes in morphology, proliferation, and migration rates and the expression of different marker proteins [22–24]. Diabetes-induced vascular complications are associated with VSMC phenotypic modulation, which involves switching from a contractile to a synthetic-proliferative phenotype [25]. Diabetes induces organic stenosis in coronary arteries, but VSMC phenotypic changes may suppress vascular hyper-contractility. Although diabetes may cause a thick plaque cap, stabilizing plaque rupture, coronary stenosis is gradually augmented. In the design of this study, we did not include insulin resistance data (such as homeostasis model assessment of insulin resistance [HOMA-IR]). Insulin resistance is frequently associated with endothelial dysfunction and has been proposed to play a pivotal role in cardiovascular diseases. Insulin sensitizations that target pathway-selective impairment in insulin signaling are known to improve endothelial dysfunction [26]. A cross-comparison with HOMA-IR may result in a different conclusion from that of this study based on a separate mechanism from HbA1c

BMI is known to be a risk factor for atherosclerosis [27]; however, BMI was not significant in our analysis and exhibited a lack of statistical power exhibited both organic stenosis and ACS. Although the precise reason is currently unclear, the following reasons are proposed. The study population was not severely obese, and the mean BMI in this study was much lower than that of Caucasians. It is probable that mild obesity has a low potential to cause harm, but obesity should beget other risks, such as diabetes, hypertension, and dyslipidemia. Clarification of the true contribution of obesity to IHD and heart failure is eagerly anticipated.

Among the results shown in Table 4, we found another curious result: high HDL was associated with ACS, although low HDL was unsurprisingly associated with diseased vessels. We cannot explain this result at present; however, according to a recent report, HDL function may be impaired in such a patient with ACS [28].

Patients in the current study with ACS consisted of those with unstable angina and myocardial infarction. We proposed a path modeling approach in which the ACS patients were divided into three groups, namely, UAP, STEMI and NSTEMI, to examine the contribution of MDA-LDL in more detail. As shown in Path model B, MDA-LDL was strongly associated with UAP and STEMI but not with NSTEMI. Rather, there was a weak but significant association of LDL with NSTEMI. Therefore, the risks of NSTEMI may be similar to those of organic stenosis from the perspective of dyslipidemia.

In this study, we proposed Path model C to examine the effects of drugs on the current findings. There was a significant association of MDA-LDL level with ACS and of HbA1c level and LDL with organic stenosis. Although this path modeling may not be able to fully eliminate the influence of drugs, the analysis indicated no influence of drugs on the current findings.

This study shows that for immediate risk avoidance, the MDA-LDL level is a suitable marker and possibly the more appropriate target of treatment. A potential therapeutic regimen in targeting MDA-LDL would be a use of strong statin to adequately decrease both LDL and MDA-LDL levels. Smoking cessation is an important component of this treatment [29].

There are several limitations of our study. The triglyceride level is commonly affected after a meal; such levels were omitted from our study based on the assumption that some patients probably ate shortly before their emergency admission. Thus, the impact of triglycerides is still unknown. Second, cigarette smoking and alcohol intake might have been underestimated in this study. It is probable that these two factors are linked more strongly to the onset of ACS because they often enhance the activity of coronary spasms in the Japanese [7,15,16]. Third, although we believe that the current path model provides new information, especially regarding the importance of MDA-LDL in ACS, the square of the multiple correlation coefficient was 0.086 for organic stenosis as an estimated value and 0.107 for ACS. This result indicates that there are many other factors associated with the progression of organic stenosis and the onset of ACS that should be clarified in the future.

In conclusion, the current statistical analysis showed clear differences among the risk factors between the progression of organic stenosis and the onset of ACS. Among them, the MDA-LDL level should be considered to represent a substantial risk of ACS.

## References

[1] Selvin E, Steffes MW, Zhu H, Matsushita K, Wagenknecht L, Pankow J, et al Glycated hemoglobin, diabetes, and cardiovascular risk in nondiabetic adults. The New England journal of medicine. 2010;362(9):800–11. Epub 2010/03/05. 10.1056/NEJMoa0908359 20200384 PMC2872990

[2] Franklin SS, Khan SA, Wong ND, Larson MG, Levy D. Is pulse pressure useful in predicting risk for coronary heart Disease? The Framingham heart study. Circulation. 1999;100(4):354–60. Epub 1999/07/27. 10421594 10.1161/01.cir.100.4.354

[3] Levy D, Wilson PW, Anderson KM, Castelli WP. Stratifying the patient at risk from coronary disease: new insights from the Framingham Heart Study. American heart journal. 1990;119(3 Pt 2):712–7; discussion 7. Epub 1990/03/01. 2137960 10.1016/s0002-8703(05)80050-x

[4] Caralis DG, Deligonul U, Kern MJ, Cohen JD. Smoking is a risk factor for coronary spasm in young women. Circulation. 1992;85(3):905–9. Epub 1992/03/01. 1537126 10.1161/01.cir.85.3.905

[5] Malik S, Wong ND, Franklin SS, Kamath TV, L'Italien GJ, Pio JR, et al Impact of the metabolic syndrome on mortality from coronary heart disease, cardiovascular disease, and all causes in United States adults. Circulation. 2004;110(10):1245–50. Epub 2004/08/25. 10.1161/01.CIR.0000140677.20606.0E 15326067

[6] Yagi H, Komukai K, Hashimoto K, Kawai M, Ogawa T, Anzawa R, et al Difference in risk factors between acute coronary syndrome and stable angina pectoris in the Japanese: smoking as a crucial risk factor of acute coronary syndrome. Journal of cardiology. 2010;55(3):345–53. Epub 2010/03/31. 10.1016/j.jjcc.2009.12.010 20350505

[7] Takaoka K, Yoshimura M, Ogawa H, Kugiyama K, Nakayama M, Shimasaki Y, et al Comparison of the risk factors for coronary artery spasm with those for organic stenosis in a Japanese population: role of cigarette smoking. International journal of cardiology. 2000;72(2):121–6. Epub 2000/01/26. 10646952 10.1016/s0167-5273(99)00172-2

[8] Kotani K, Maekawa M, Kanno T, Kondo A, Toda N, Manabe M. Distribution of immunoreactive malondialdehyde-modified low-density lipoprotein in human serum. Biochimica et biophysica acta. 1994;1215(1–2):121–5. Epub 1994/11/17. 7947993 10.1016/0005-2760(94)90100-7

[9] Tanaga K, Bujo H, Inoue M, Mikami K, Kotani K, Takahashi K, et al Increased circulating malondialdehyde-modified LDL levels in patients with coronary artery diseases and their association with peak sizes of LDL particles. Arteriosclerosis, thrombosis, and vascular biology. 2002;22(4):662–6. Epub 2002/04/16. 11950707 10.1161/01.atv.0000012351.63938.84

[10] Shigematsu S, Takahashi N, Hara M, Yoshimatsu H, Saikawa T. Increased incidence of coronary in-stent restenosis in type 2 diabetic patients is related to elevated serum malondialdehyde-modified low-density lipoprotein. Circulation journal: official journal of the Japanese Circulation Society. 2007;71(11):1697–702. Epub 2007/10/30. 17965487 10.1253/circj.71.1697

[11] Falk E, Shah PK, Fuster V. Coronary plaque disruption. Circulation. 1995;92(3):657–71. Epub 1995/08/01. 7634481 10.1161/01.cir.92.3.657

[12] Fuster V, Badimon L, Badimon JJ, Chesebro JH. The pathogenesis of coronary artery disease and the acute coronary syndromes (1). The New England journal of medicine. 1992;326(4):242–50. Epub 1992/01/23. 10.1056/NEJM199201233260406 1727977

[13] Libby P. Molecular bases of the acute coronary syndromes. Circulation. 1995;91(11):2844–50. Epub 1995/06/01. 7758192 10.1161/01.cir.91.11.2844

[14] Libby P. Mechanisms of acute coronary syndromes and their implications for therapy. The New England journal of medicine. 2013;368(21):2004–13. Epub 2013/05/24. 10.1056/NEJMra1216063 23697515

[15] Mizuno Y, Harada E, Morita S, Kinoshita K, Hayashida M, Shono M, et al East asian variant of aldehyde dehydrogenase 2 is associated with coronary spastic angina: possible roles of reactive aldehydes and implications of alcohol flushing syndrome. Circulation. 2015;131(19):1665–73. Epub 2015/03/12. 10.1161/CIRCULATIONAHA.114.013120 25759460

[16] Yasue H, Nakagawa H, Itoh T, Harada E, Mizuno Y. Coronary artery spasm—clinical features, diagnosis, pathogenesis, and treatment. Journal of cardiology. 2008;51(1):2–17. Epub 2008/06/05. 10.1016/j.jjcc.2008.01.001 18522770

[17] Maseri A, Beltrame JF, Shimokawa H. Role of coronary vasoconstriction in ischemic heart disease and search for novel therapeutic targets. Circulation journal: official journal of the Japanese Circulation Society. 2009;73(3):394–403. Epub 2009/02/10. 19202303 10.1253/circj.cj-09-0033

[18] Kinoshita K, Kawai M, Minai K, Ogawa K, Inoue Y, Yoshimura M. Potent influence of obesity on suppression of plasma B-type natriuretic peptide levels in patients with acute heart failure: An approach using covariance structure analysis. International journal of cardiology. 2016;215:283–90. Epub 2016/04/30. 10.1016/j.ijcard.2016.04.111 27128547

[19] Thygesen K, Alpert JS, Jaffe AS, Simoons ML, Chaitman BR, White HD. Third universal definition of myocardial infarction. Global heart. 2012;7(4):275–95. Epub 2012/12/01. 10.1016/j.gheart.2012.08.001 25689940

[20] Feletou M, Vanhoutte PM. Endothelial dysfunction: a multifaceted disorder (The Wiggers Award Lecture). American journal of physiology Heart and circulatory physiology. 2006;291(3):H985–1002. Epub 2006/04/25. 10.1152/ajpheart.00292.2006 16632549

[21] Bekkering S, Quintin J, Joosten LA, van der Meer JW, Netea MG, Riksen NP. Oxidized low-density lipoprotein induces long-term proinflammatory cytokine production and foam cell formation via epigenetic reprogramming of monocytes. Arteriosclerosis, thrombosis, and vascular biology. 2014;34(8):1731–8. Epub 2014/06/07. 10.1161/ATVBAHA.114.303887 24903093

[22] Aikawa M, Yamaguchi H, Yazaki Y, Nagai R. Smooth muscle phenotypes in developing and atherosclerotic human arteries demonstrated by myosin expression. Journal of atherosclerosis and thrombosis. 1995;2(1):14–23. Epub 1995/01/01. 9225203 10.5551/jat1994.2.14

[23] Psaltis PJ, Simari RD. Vascular wall progenitor cells in health and disease. Circulation research. 2015;116(8):1392–412. Epub 2015/04/11. 10.1161/CIRCRESAHA.116.305368 25858065

[24] Potter CM, Lao KH, Zeng L, Xu Q. Role of biomechanical forces in stem cell vascular lineage differentiation. Arteriosclerosis, thrombosis, and vascular biology. 2014;34(10):2184–90. Epub 2014/07/12. 10.1161/ATVBAHA.114.303423 25012135

[25] Moretto P, Karousou E, Viola M, Caon I, D'Angelo ML, De Luca G, et al Regulation of hyaluronan synthesis in vascular diseases and diabetes. Journal of diabetes research. 2015;2015:167283 Epub 2015/04/03. 10.1155/2015/167283 25834831 PMC4365328

[26] Muniyappa R, Sowers JR. Role of insulin resistance in endothelial dysfunction. Reviews in endocrine & metabolic disorders. 2013;14(1):5–12. Epub 2013/01/12. 23306778 10.1007/s11154-012-9229-1 PMC3594115

[27] Mora S, Yanek LR, Moy TF, Fallin MD, Becker LC, Becker DM. Interaction of body mass index and framingham risk score in predicting incident coronary disease in families. Circulation. 2005;111(15):1871–6. Epub 2005/04/20. 10.1161/01.CIR.0000161956.75255.7B 15837938

[28] Annema W, Willemsen HM, de Boer JF, Dikkers A, van der Giet M, Nieuwland W, et al HDL function is impaired in acute myocardial infarction independent of plasma HDL cholesterol levels. Journal of clinical lipidology. 2016;10(6):1318–28. Epub 2016/12/07. 10.1016/j.jacl.2016.08.003 27919348

[29] Ogawa K, Tanaka T, Nagoshi T, Sekiyama H, Arase S, Minai K, et al Increase in the oxidised low-density lipoprotein level by smoking and the possible inhibitory effect of statin therapy in patients with cardiovascular disease: a retrospective study. BMJ open. 2015;5(1):e005455 Epub 2015/01/23. 10.1136/bmjopen-2014-005455 25609666 PMC4305066

